# Autonomic small fiber involvement in painful long COVID: a histological and clinical study

**DOI:** 10.3389/fnhum.2025.1719705

**Published:** 2026-01-14

**Authors:** Pietro Falco, Eleonora Galosi, Daniel Litewczuk, Enrico Evangelisti, Giulia Di Stefano, Lars Arendt-Nielsen, Andrea Truini, Caterina Maria Leone

**Affiliations:** 1 Department of Human Neuroscience, Sapienza University, Rome, Italy; 2 Centre for Neuroplasticity and Pain, Department of Health Science and Technology, School of Medicine, Aalborg University, Aalborg, Denmark; 3 Department of Gastroenterology and Hepatology, Mech-Sense, Clinical Institute, Aalborg University Hospital, Aalborg, Denmark; 4 Steno Diabetes Center North Denmark, Clinical Institute, Aalborg University Hospital, Aalborg, Denmark

**Keywords:** autonomic dysfunction, long COVID, pain, skin biopsy, small fiber neuropathy (SFN)

## Abstract

**Background:**

Despite growing recognition of painful long COVID syndrome as a chronic neurological condition marked by pain and autonomic symptoms, the precise contribution of autonomic small fiber involvement is still not well characterized and understood. In this retrospective study, we aimed to identify autonomic small fiber involvement in patients with painful long COVID syndrome by analyzing skin biopsy data. We used nerve fiber density in the piloerector muscles (PMNFD) and sweat glands (SGNFD) as the primary histological outcomes of autonomic innervation.

**Methods:**

We reviewed skin biopsy samples from 50 patients with painful long COVID syndrome and included 31 patients with available PMNFD and SGNFD data for analysis. PMNFD and SGNFD were compared with an age- and sex-matched healthy control group (*n* = 42). To evaluate whether autonomic involvement was independent of somatic nerve fiber reduction, a subgroup analysis was performed in patients with normal intraepidermal nerve fiber density (IENFD) (*n* = 23). Correlations between histological findings and autonomic symptoms, assessed with the COMPASS-31 questionnaire, were also analyzed.

**Results:**

Piloerector muscle nerve fiber density and SGNFD were significantly reduced in patients with long COVID compared with controls, both in the full sample (*p* = 0.0135; *p* < 0.0001) and in the subgroup with normal IENFD (*p* = 0.0003; *p* = 0.0005). Neither PMNFD nor SGNFD correlated with COMPASS-31 scores (*p* = 0.27; *p* = 0.46) and no association with disease onset, duration and COVID-19 severity was found.

**Conclusion:**

These findings provide histological evidence that autonomic small fiber damage is a prominent and measurable feature of painful long COVID syndrome. Importantly, this pathology was also observed in patients with preserved IENFD, indicating that autonomic involvement may occur independently of somatic small fiber loss.

## Introduction

Since its emergence, coronavirus disease 2019 (COVID-19) has been associated not only with acute respiratory manifestations but also with long-term sequelae involving multiple organ systems with up to 200 different symptoms reported (World Health Organization [WHO], 2024). Post-acute sequelae of SARS-CoV-2 infection (PASC), commonly referred to as long COVID, have increasingly been recognized as a heterogeneous clinical syndrome, affecting approximately 10–30% of individuals following acute infection (Fernández-de-las-Peñas et al., 2024; Soriano et al., 2022). Persistent pain and autonomic symptoms are among the most frequent and disabling manifestations (Di Stefano et al., 2023; Kuodi et al., 2023).

Several studies have reported autonomic dysfunction in patients with long COVID who present with autonomic symptoms, including high autonomic symptom burden on COMPASS-31 (Eastin et al., 2025), cardiovascular autonomic testing abnormalities (Marques et al., 2023; Reis Carneiro et al., 2023), and orthostatic intolerance at tilt-table evaluations (Jamal et al., 2022). Autonomic reflex screens including QSART have also identified sudomotor abnormalities in subsets of patients (Novak et al., 2022). However, these findings derive primarily from functional assessments, which can be influenced by central nervous system modulation.

Recent evidence also suggests that small fiber neuropathy (SFN)—typically associated with neuropathic pain and autonomic complaints—may be present in a subset of patients (Falco et al., 2024b; Oaklander et al., 2022). However, SFN alone does not account for the autonomic symptoms observed in the majority of cases and the morphological substrate of autonomic dysfunction in long COVID remains limited and incompletely characterized (Donadio et al., 2025).

Skin biopsy offers the unique opportunity to evaluate both somatic and autonomic innervation. Beyond intraepidermal nerve fiber density (IENFD), which reflects somatic sensory innervation of the epidermis, it also enables the quantification of sympathetic fibers innervating dermal structures such as the piloerector muscle and the sweat gland. Piloerector muscle nerve fiber density (PMNFD) and sweat gland nerve fiber density (SGNFD) are increasingly recognized as valuable markers of autonomic integrity (Lauria et al., 2010; Luo et al., 2011; Nolano et al., 2010).

Exploring structural changes in dermal autonomic innervation, and their relationship with patient-reported autonomic symptoms, could provide important insights into the extent and clinical relevance of autonomic small fiber involvement in long COVID.

In this study, we retrospectively analyzed skin biopsy samples from a cohort of patients with painful long COVID, focusing on PMNFD and SGNFD as primary markers of autonomic involvement, and compared them with healthy controls. Our aim was to determine whether these patients exhibit evidence of autonomic small fiber damage, to assess whether such damage occurs independently of somatic small fiber loss, and to establish its clinical relevance by examining the relationship between morphological alterations and patient-reported symptoms.

## Materials and methods

### Study cohort and design

We retrospectively reviewed skin biopsy data collected in patients with suspected small fiber impairment at the Peripheral Neuropathy and Neuropathic Pain Unit, Department of Human Neuroscience, Sapienza University, Rome, between January 2022 and March 2025, and selected patients diagnosed with painful long COVID syndrome complaining of autonomic symptoms. Half of the enrolled patients have already been included in a previous study on the assessment of SFN in the context of painful long COVID (Falco et al., 2024b).

All patients had a confirmed history of SARS-CoV-2 infection documented by nasopharyngeal polymerase chain reaction. Inclusion criteria were: age > 18 years; onset of pain and autonomic symptoms within 3 months after SARS-CoV-2 infection; persistence of these symptoms for at least 6 months; normal large-fiber function as determined by neurological examination and standard nerve conduction studies. Exclusion criteria were: pre-existing chronic pain disorders; concurrent central or peripheral nervous system diseases; conditions known to affect the peripheral nervous system (e.g., diabetes, vitamin B12 deficiency, renal failure, autoimmune disorders, or prior exposure to neurotoxic therapies); psychiatric disorders; inability to provide informed consent.

All included patients underwent a comprehensive clinical evaluation, including detailed medical history, nerve conduction studies, and skin biopsy at the distal calf. Demographic and clinical data were reviewed, and at the time of clinical assessment all patients received a comprehensive laboratory work-up to exclude potential causes of small fiber neuropathy, including metabolic screening (fasting plasma glucose, glycated hemoglobin, lipid profile), vitamin B12 and folate levels, renal and liver function tests, thyroid function tests, rheumatologic screening, and infectious disease screening.

The primary morphological outcome measures of autonomic small fibers were piloerector muscle nerve fiber density (PMNFD) and sweat gland nerve fiber density (SGNFD) at the distal biopsy site in patients with painful long COVID. These were compared with a group of 42 age- and sex-matched healthy controls. To determine whether autonomic involvement occurred independently of somatic small fiber damage, a subgroup analysis was performed in patients with normal IENFD. Morphological findings were correlated to autonomic symptoms as assessed by the COMPASS-31 questionnaire.

The study was conducted in accordance with the Declaration of Helsinki and approved by the local institutional review board (0059/2022); written informed consent was obtained from all participants.

### Clinical evaluation

All subjects included in the study underwent detailed medical history recording and neurological examination. COVID-19–related variables, including COVID-19 disease severity classified according to international guidelines (COVID-19 Treatment Guidelines Panel, 2024), the interval between acute infection and the onset of symptoms (disease onset), and the time elapsed from symptom onset to skin biopsy (disease duration) were collected. Autonomic symptoms were assessed with the 31-item Composite Autonomic Symptom Score (COMPASS-31), a structured interview including questions related to the presence and severity of orthostatic intolerance, vasomotor alterations (skin changes), secretomotor symptoms (dry mouth, dry eyes, and abnormal sweating), gastrointestinal disturbances, genitourinary symptoms, and pupillomotor abnormalities. We collected the total COMPASS-31 score and the 5 subscores related to the main categories of dysautonomia symptoms: orthostatic intolerance, vasomotor, secretomotor, gastrointestinal, bladder, and pupillomotor symptoms (Pierangeli et al., 2015). Painful symptoms were assessed with the Neuropathic Pain Symptom Inventory (NPSI) questionnaire, a validated questionnaire commonly used for scoring the different types of neuropathic pain. The NPSI was administered with reference to the distally distributed pain area, as this region was consistently reported as painful by all patients. We calculated the NPSI subscores for the different pain qualities, including ongoing pain (burning and pressing pain), paroxysmal pain, provoked pain, and abnormal sensation (paraesthesia and dysesthesias) (Bouhassira et al., 2004).

Touch and pinprick perception were tested using cotton wool and a wooden cocktail stick. For the cold detection, a metal rod at 22 °C room temperature was applied for 3 s. For the warm detection, a glass vial was applied for 3 s to the skin after having been warmed within the examiner’s hands at body temperature (36–37 °C). Vibration perception was measured with a 128 Hz graduated Rydel–Seiffer tuning fork. Patients were instructed to identify any pain caused by normally non-painful touch (allodynia) or an exaggerated pain response to pinprick stimuli (hyperalgesia). Deep tendon reflexes were assessed and graded as normal, decreased, or absent. We used the Medical Research Council (MRC) score to grade muscle strength (Cruccu et al., 2010).

### Nerve conduction study

Nerve conduction studies (NCS) were performed in all patients using surface electrodes, following the recommendations of the International Federation of Clinical Neurophysiology. Sensory nerve action potentials and conduction velocities were recorded from the sural, ulnar, and superficial radial nerves; compound motor action potentials and conduction velocities were measured in the peroneal, tibial, and ulnar nerves. Skin temperature was maintained between 34 °C and 36 °C. Results were compared to age-adjusted normative values (Stålberg et al., 2019).

### Skin biopsy

Patients underwent skin biopsy at the distal leg, 10 cm above the lateral malleolus, using a 3 mm disposable circular punch after local lidocaine anaesthesia. Biopsies were performed under sterile conditions and with no suture required. Using indirect immunofluorescence, intraepidermal innervation was assessed with the pan-neuronal marker Protein Gene Product 9.5 (PGP9.5). Biopsies were fixed for 24 h at 4 °C in Zamboni’s fixative, then were cryoprotected overnight with a solution containing 30% (v/v) ethylene glycol and 30% (w/v) sucrose in phosphate-buffered saline with 1% polyvinylpyrrolidone (PVP). A cut was performed at -23 °C with a cryostat (MEV, SLEE medical GmbH, Nieder- Olm, Germany) to obtain 50 μm-thick sections. Three non-consecutive free-floating sections were randomly selected for immunostaining from each sample and blocked with 5% normal donkey serum for 1 h. Sections were then incubated overnight with a rabbit anti-human PGP9.5 monoclonal antibody (Abcam, Cambridge, UK, 1:500 diluted) and a mouse antihuman collagen IV monoclonal antibody (Millipore, Darmstadt, Germany, 1:1600). The following day, sections were incubated with anti-rabbit-Cy3 (Jakson ImmunoResearch, Cambridgeshire, UK, 1:800) and anti-mouse-488 (Jakson ImmunoResearch, Cambridgeshire, UK, 1:400) secondary antibodies overnight.

We calculated IENFD according to European Federation of Neurological Societies and Peripheral Nerve Society guidelines (Lauria et al., 2010). Epidermal linear length was measured through (NIS AR software, Nikon, Tokyo, Japan) to obtain a linear density (number of fibers/mm). Normative values from an internationally recognized dataset were used (Provitera et al., 2016).

We calculated piloerector muscle nerve fiber density (PMNFD) on all piloerector muscles available in the sections. We included in this study only participants with at least 3 distinct piloerector muscles in the sections selected. For each piloerector muscle, a photo was taken at the level of maximum diameter, focusing on as many fibers as possible. To quantify the innervation, a vertical line to the nerve fibers was drawn, and fibers intersecting the line were counted. Nerve fiber density for each piloerector muscle was calculated as the ratio between the number of nerve fibers crossing the line in focus and the width of the line (nerve fibers/mm). For each participant, piloerector muscles nerve fiber density corresponded to the average nerve fiber density of the 3 most innervated piloerector muscles (Nolano et al., 2010). We evaluated SGNFD with a semiquantitative approach for all sweat glands available in the sections, as previously described. For each sweat gland, we collected a photo at the level of the maximum diameter of the sweat gland, focusing on as many fibers as possible. Using a 0 to 4 scale, we applied a semiquantitative score of sweat gland innervation. A score of 0 indicated that no nerve fiber was identifiable, 1 corresponded to severely, 2 to moderately, 3 to mildly reduced nerve fiber density, and 4 to normal nerve fiber density. We included in the analysis only participants with at least 3 evaluable sweat glands. We then assigned a 0 to 4 score to each participant, corresponding to the mean density of all analyzed glands in the samples (Falco et al., 2024a; Luo et al., 2011).

All skin biopsies were collected and processed in the laboratory of the Peripheral Neuropathy and Neuropathic Pain Unit at the Department of Human Neuroscience, Sapienza University. Two blinded investigators (P.F. and E.G.) calculated PMNFD and SGNFD with a fluorescence microscope (Nikon Eclipse, Nikon, Tokyo, Japan) with appropriate wavelength filters. The average nerve fiber density calculated by the 2 operators (both for piloerector muscle and sweat gland innervation) was used as outcome variable. PMNFD and SGNFD values of patients were then compared to those of 42 sex and age-matched healthy participants. The same operators blindly analyzed 30 external biopsies prior to the study; Cronbach’s alpha testing showed good inter-operator agreement for both piloerector muscle (α = 0.84) and SGNFD (α = 0.76) quantification.

Patients with reduced IENFD and at least two clinical signs were considered small fiber neuropathy, according to Besta criteria (Devigili et al., 2019).

### Statistical analysis

A descriptive analysis was performed to summarize the main demographic, clinical, and diagnostic test variables in patients with painful Long COVID. The results are reported as mean ± standard deviation (SD) for continuous variables and frequency for categorical variables. Normality distribution was verified using the Shapiro-Wilk normality test. Accordingly, we used the non-parametric Mann-Whitney test to compare autonomic skin biopsy variables at distal sites between patients with painful Long COVID and healthy control group. To determine whether autonomic damage was independent of somatic small fiber involvement, we performed a subgroup analysis comparing autonomic skin biopsy measures in long COVID patients with normal IENFD to those of healthy controls. We used Spearman’s test to analyze the bivariate relationships between the autonomic skin biopsy variables and the COMPASS-31. Additionally, a correlation was performed between autonomic skin biopsy and clinical variables (disease duration, disease onset and COVID-19 disease severity). Prism 9.5 (GraphPad Software, Boston, MA) was adopted for statistical analysis and a *P*-value < 0.05 was considered statistically significant.

## Results

Skin biopsy data from 50 patients with painful long COVID were screened. Of these, 31 had valuable piloerector muscles and sweat glands in distal skin biopsy samples and were therefore included in the analysis. Demographic and clinical characteristics of the included patients are summarized in Table 1. The most frequent autonomic symptoms were orthostatic intolerance (77%), followed by gastrointestinal (52%) and secretomotor symptoms (52%). According to the NPSI questionnaire, burning pain, pins and needles and tingling paraesthesia were the most frequently reported pain qualities (Supplementary Table 1). The time from SARS-CoV-2 infection to the onset of painful and autonomic symptoms ranged from 0 to 2 months, with 6 patients (19%) presenting with an acute onset during the infection (16.32 ± 20.93 dd). The mean duration of disease was 15.35 ± 11.5 months, and the large majority of patients (74%) had a low COVID-19 disease severity score (score = 1). Of the 31 patients, 22 (71%) had received an mRNA-based SARS-CoV-2 vaccination prior to contracting COVID-19. The last vaccine dose in these patients was administered between 4 and 12 months before infection. At the time of evaluation, 12 patients were receiving pain medication but continued to report symptoms, with 7 using gabapentinoids and 5 antidepressants. All patients showed no evidence of large fiber involvement on neurological examination, and nerve conduction studies were within normal limits. Twenty- three patients showed normal IENFD at skin biopsy, while 8 had reduced IENFD. All the patients with reduced IENFD fulfilled the Besta criteria for SFN.

**TABLE 1:** Demographic, clinical and QST parameters of patients with painful long-COVID.

	Painful long COVID patients (*N* = 31)	Controls (*n* = 42)
Age (years)	49.4 (13.72)	49.29 (14.54)
Sex (M:F)	13:18	18:24
BMI	25.83 (4.65)	24.41 (4.04)
COVID-19 severity*	Mild (23), moderate (5), severe (2), critical (1)	
Onset of symptoms (days)**	16.32 (20.93)	
Disease duration (months)***	15.35 (11.51)	
COMPASS-31 score	21.76 (13.38)	
NPSI score	24.46 (18.75)	

Each value is expressed as mean (SD) or frequency for categorical variable.

*According to the clinical spectrum of SARS-CoV-2 infection from National Institutes of Health COVID-19 Treatment Guideline.

**Time in days from COVID-19 to sensory symptoms’ onset.

***Time in months from symptoms onset to visit. COMPASS 31: Composite Autonomic Symptom Score.

**FIGURE 1:**
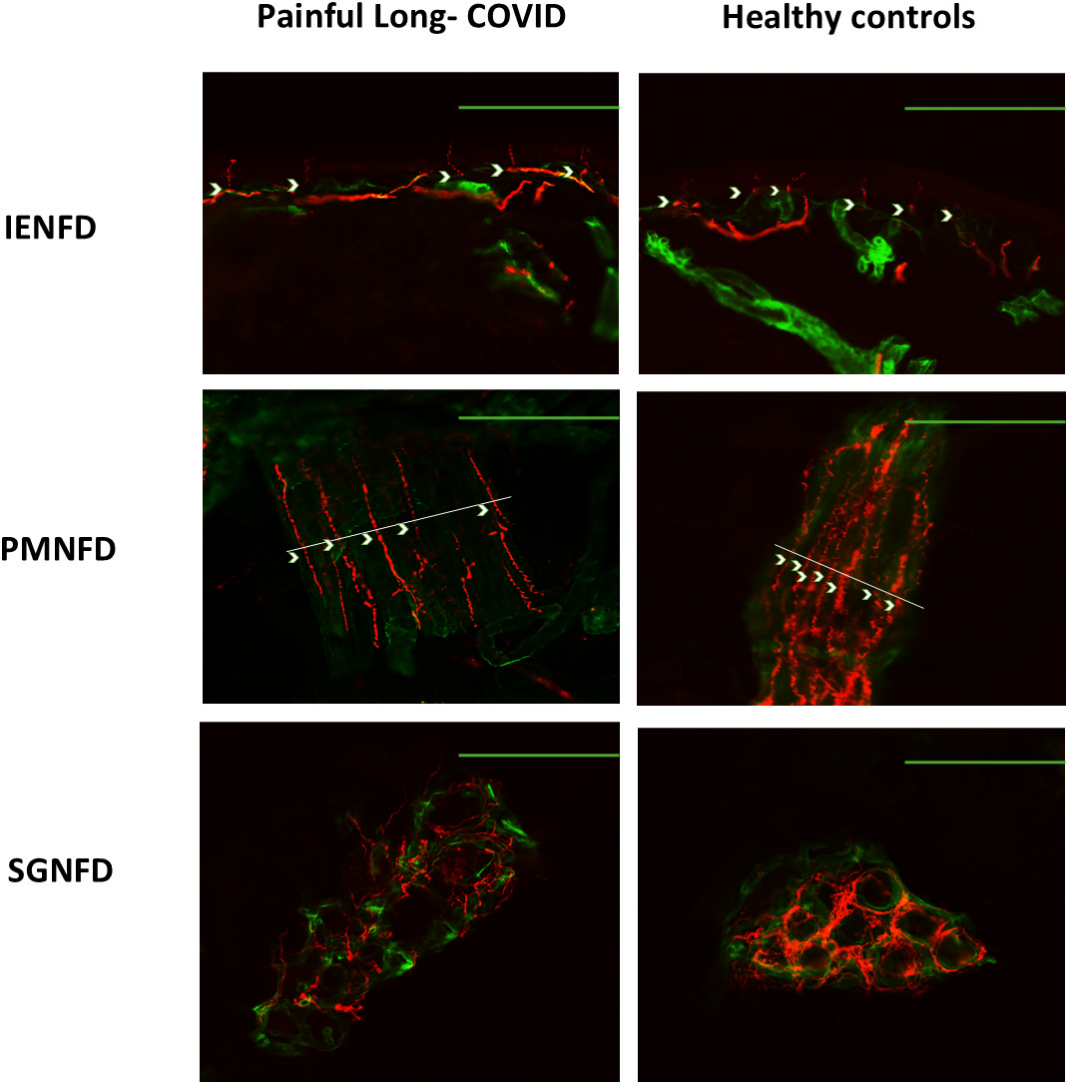
Representative skin biopsy images from a subgroup of patients with painful long COVID showing normal IENFD, and the sex- and age-matched healthy controls. Examples of nerve fiber quantification are provided for intraepidermal nerve fiber density (IENFD), piloerector muscle nerve fiber density (PMNFD), and sweat gland nerve fiber density (SGNFD). Red staining represents PGP9.5 for nerve fibers, while green staining corresponds to collagen IV. Autonomic fiber densities (PMNFD and SGNFD) were reduced in patients with painful long COVID compared with healthy controls, whereas no differences were observed in IENFD. IENFD calibration bars: 200 μm; piloerector muscle and sweat gland nerve fiber density calibration bars: 100 μm.

**FIGURE 2:**
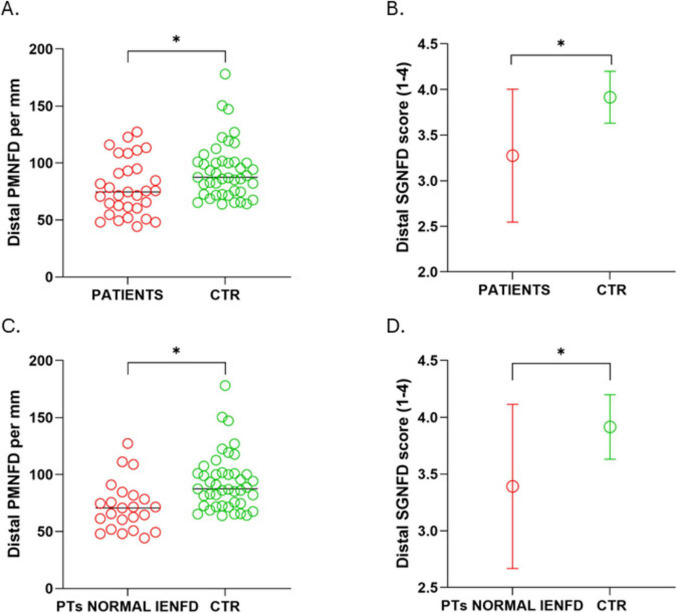
Reduced PMNFD and SGNFD in patients with long COVID compared to controls. **(A)** Individual values of PMNFD of all patients versus controls; **(B)** mean and standard deviation of SGNFD of all patients versus controls; **(C)** individual values of PMNFD of patients with normal IENFD versus controls; **(D)** mean and standard deviation of SGNFD of patients with normal IENFD versus controls. *p < 0.05.

Both PMNFD and SGNFD at the distal site were significantly reduced in painful long COVID patients compared with age- and sex-matched healthy controls (*p* = 0.0118 and *p* < 0.0001, respectively) (Table 2). In the subgroup analysis restricted to patients with normal IENFD, reductions in both PMNFD and SGNFD at the distal site remained significant compared with healthy controls (*p* = 0.0003 and *p* = 0.0017) (Figures 1, 2 and Table 2). No correlation was found for neither of the two morphological autonomic outcomes variables (PMNFD and SGNFD) and the total score of the COMPASS-31 questionnaire (*r* = 0.23, *p* = 0.272; *r* = −0.02, *p* = 0.464). No association was found between PMNFD and SGNFD and disease duration (*r* = −0.06; *p* = 0.723), disease onset (*r* = −0.3; *p* = 0.114) and COVID-19 disease severity (*r* = −0.23; *p* = 0.21).

**TABLE 2:** Comparison of skin biopsy parameters between patients with painful long COVID and healthy controls.

	Painful long COVID patients (*N* = 31)	Healthy controls (*N* = 42)	*p*
PMNFD	78.44 (24.42)	93.15 (24.93)	**0.0118**
SGNFD	3.27 (0.73)	3.91 (0.28)	**<0.0001**
PMNFD (subgroup with normal IENFD*)	71.87 (21.69)	93.15 (24.93)	**0.0003**
SGNFD (subgroup with normal IENFD*)	3.5 (0.64)	3.91 (0.28)	**0.0017**

Each value is expressed as mean (SD). *n = 23. PMNFD, piloerector muscle nerve fiber density; SGNFD, sweat gland nerve fiber density. *P*-values reaching statistical significance are highlighted in bold.

## Discussion

In this skin biopsy and clinical study, we show that patients with pain and autonomic symptoms associated with long COVID exhibit reduced small nerve fiber density in dermal autonomic- innervated annexes, i.e., piloerector muscles and sweat glands. Notably, this autonomic fiber damage was also observed in individuals with normal IENFD, suggesting that autonomic involvement in long COVID may occur independently of somatic small fiber loss.

Previous studies have shown that a considerable proportion of patients with autonomic symptoms associated with long COVID display abnormalities on functional autonomic testing, such as cardiovascular autonomic tests and QSART (Eastin et al., 2025; Jamal et al., 2022; Marques et al., 2023; Novak et al., 2022; Reis Carneiro et al., 2023). A small study also demonstrated cardiac sympathetic denervation using 123I-MIBG SPECT (Xavier de Brito et al., 2024). Our findings provide direct morphological confirmation that patients with long COVID presenting with pain and autonomic symptoms display autonomic small fiber pathology.

These results partly differ from those of a recent study reporting no differences in autonomic innervation between 33 patients with burning pain and autonomic symptoms following COVID-19 and healthy controls (Donadio et al., 2025). Such discrepancies may be related to differences in the timing of skin biopsy acquisition between the two studies, with a longer disease duration in our cohort compared with the aforementioned study, as well as to differences in patient inclusion criteria. An additional source of variability may be related to the different techniques used for the quantification of dermal autonomic fibers. In the study by Donadio et al. (2025) PMNFD and SGNFD were quantified using an automated approach, whereas in the present study we employed manual counting for PMNFD (Nolano et al., 2010) and a semiquantitative method for SGNFD. At present, no international standard is available for the histological quantification of dermal autonomic small fibers using immunofluorescence. While sympathetic fibers innervating the piloerector muscle display a relatively linear and regular course that allows reliable manual quantification, sweat gland fibers exhibit a more complex architecture and distribution. We believe that a semiquantitative approach to SGNFD assessment allows a comprehensive evaluation of sympathetic innervation, accounting for fiber distribution and regularity, with good intra-operator agreement.

Neuropathic pain and autonomic symptoms frequently coexist in patients with long COVID. Consistent with prior work—including our own—SFN has been identified in a substantial subset of patients with long COVID (Donadio et al., 2025; Falco et al., 2024b; Oaklander et al., 2022; Novak et al., 2022). Nevertheless, the presence of SFN *per se*, as defined by recognized criteria, does not account for the near-universal autonomic symptomatology observed in our cohort. In our study, we observed histological evidence of postganglionic sympathetic fibers damage in dermal structures even in patients with normal IENFD, indicating that autonomic involvement can occur independently of SFN. These findings support incorporating targeted histological evaluation of autonomic fibers—beyond standard SFN work-up—into the diagnostic assessment of long-COVID dysautonomia.

Several mechanisms have been proposed to explain small fiber involvement in long COVID, including immune-mediated injury, persistent inflammatory activity, microvascular dysfunction, and direct viral neurotropism (McFarland et al., 2021; Taga and Lauria, 2022). The presence of autoantibodies targeting autonomic receptors may lead to both functional dysregulation and structural injury of sympathetic fibers, while preclinical data demonstrate that SARS-CoV-2 can directly infect sensory and autonomic neurons expressing hACE2, suggesting a potential neurotropic effect (Joyce et al., 2024). Our finding of reduced dermal autonomic fiber density, even in patients with preserved IENFD, is consistent with these hypotheses and supports the notion that autonomic fibers may represent a preferential target of COVID-19–related injury.

In our cohort, we did not observe correlations between autonomic morphological parameters and clinical measures of autonomic symptoms, consistent with a previous report (Falco et al., 2024a). This finding underscores that patient-reported symptoms are largely modulated at the central nervous system level, and that additional factors such as psychosocial stress, mood disturbances, and sleep disorders are likely to contribute to symptom amplification. Furthermore, COMPASS-31, while highly useful for detecting and grading autonomic symptoms, is not designed to index the anatomical substrate or polarity of autonomic dysfunction, nor to map directly onto skin-biopsy morphometrics; it yields a weighted, multidomain symptom score that integrates both central and peripheral drivers.

In our cohort, neither of the two morphological autonomic outcomes showed significant associations with disease duration, time of onset, or COVID-19 clinical severity. These data suggest that peripheral autonomic injury in long COVID may follow a non-linear “hit-and-run” trajectory, characterized by early damage that plateaus, such that cross-sectional morphology no longer reflects time since onset. It should be noted that our ability to detect associations may be constrained by the small cohort and the skewed severity distribution, with most participants exhibiting low global long-COVID severity.

Further studies with larger cohorts and longitudinal designs are needed to validate these results, elucidate the underlying pathogenic mechanisms, and assess their prognostic and therapeutic implications.

### Limitations

This study has several limitations. First, its retrospective design and relatively small sample size may restrict the generalizability of our findings. Second, although skin biopsy provides direct histological evidence, the semiquantitative approach employed for SGNFD may introduce variability; however, the use of blinded raters helps to mitigate this limitation. Third, referral bias cannot be excluded, as patients with painful long COVID were specifically recruited on the basis of post–COVID-19 painful and autonomic complaints. In addition, the absence of a control group consisting of individuals with documented COVID-19 who did not develop long COVID–related symptoms represents a further limitation. Finally, although we observed a close temporal association between COVID-19 and the onset of symptoms, the study design does not allow a definitive causal relationship to be established. Additionally, over-representation of lower-severity phenotypes limits external validity, as results may not generalize to patients with moderate–severe long-COVID dysautonomia.

## Conclusion

In conclusion, this study provides histological evidence that autonomic postganglionic small fiber damage occurs in patients with painful long COVID and may develop independently of somatic small fiber involvement, although no association was found with patient-reported symptoms. These findings suggest that autonomic fibers may represent a specific target of COVID-19–related injury.

## Data Availability

The datasets presented in this article are not readily available because data are available on reasonable request from the authors. Requests to access the datasets should be directed to PF, pietro.falco@uniroma1.it.
