# Supplementary material for: Autonomic small fiber involvement in painful long COVID: a histological and clinical study
Source: Front Hum Neurosci. 2026 Jan 14;19:1719705. doi: 10.3389/fnhum.2025.1719705 (PMC12847426; doi:10.3389/fnhum.2025.1719705)
Supplement: Supplementary file 1 [file Table_1.docx]

**Supplementary Table 1.** NPSI and COMPASS-31 sub-domains data of the painful Long COVID patients.

| NPSI item | Frequency (%) | Severity*  (mean ± SD) |
| --- | --- | --- |
| Q1 burning pain | 45 | 4.2±3.9 |
| Q2 squeezing pain | 35 | 3.0±3.5 |
| Q3 pressure pain | 29 | 2.5±3.5 |
| Q5 electric shocks | 29 | 2.7±3.9 |
| Q6 stabbing pain | 23 | 2.1±3.6 |
| Q8 pain provoked by brushing | 16 | 1.4±2.8 |
| Q9 pain provoked by pressure | 13 | 1.1±2.5 |
| Q10 pain provoked by cold | 10 | 0.8±2.2 |
| Q11 pins and needles paraesthesia | 39 | 3.1±3.5 |
| Q12 Tingling paraesthesia | 55 | 4.2±3.3 |
| Orthostatic intolerance | 77 | 13.2±9.7 |
| Vasomotor | 26 | 0.8±1.3 |
| Secretomotor | 52 | 3±2.9 |
| Gastrointestinal | 52 | 3.6±3.7 |
| Bladder | 26 | 0.4±0.9 |
| Pupillomotor | 39 | 0.7±0.9 |

NPSI: Neuropathic Pain Symptom Inventory; COMPASS 31: Composite Autonomic Symptom Score

*Severity is expressed as 0-10 score for each NPSI sub-domain and according to the different weighted score for each COMPASS-31 sub-domain (Orthostatic intolerance: 0-40; Vasomotor 0-5; Secretomotor 0-15; Gastrointestinal 0-25; Bladder 0-10; Pupillomotor 0-5).
